# Supplementary material for: Polyketide synthase gene clusters in the ericoid mycorrhizal fungus Oidiodendron maius: genome-wide prediction, expression profiling, and functional characterization of knockout mutants
Source: Front Plant Sci. 2026 Sep 16;17:1924430. doi: 10.3389/fpls.2026.1924430 (PMC13623903; doi:10.3389/fpls.2026.1924430)
Supplement: Supplementary file 1 [file SupplementaryFile1.zip › Supplementary Materials/Supplementary Folder 1.zip!/Oidma1_AssemblyScaffolds_Repeatmasked/knownclusterblast/cluster23/jgi.p_Oidma1_125578_mibig_hits.html]

| MIBiG Protein | Description | MIBiG Cluster | MiBiG Product | % ID | % Coverage | BLAST Score | E-value |
| --- | --- | --- | --- | --- | --- | --- | --- |
| AMY15055.1 | MFS\_transporter | BGC0001339 | Squalestatin\_S1\_biosynthetic\_gene\_cluster | 51 | 97.8300180832 | 550 | 2.7e-156 |
| ACS68556.1 | major\_facilitator\_superfamily\_protein | BGC0001026 | NG-391\_biosynthetic\_gene\_cluster | 37 | 88.6075949367 | 313 | 5.6e-85 |
| BAE56594.1 |  | BGC0001123 | Aspirochlorine\_biosynthetic\_gene\_cluster | 33 | 96.2025316456 | 287 | 3.3e-77 |
| CAF31575.1 | putative\_kanamycin\_efflux\_protein | BGC0000705 | Kanamycin\_biosynthetic\_gene\_cluster | 30 | 90.7775768535 | 233 | 4.3e-61 |
| CAF60521.1 | putative\_efflux\_protein | BGC0000704 | Kanamycin\_biosynthetic\_gene\_cluster | 30 | 90.7775768535 | 233 | 4.3e-61 |
| EAU38977.1 | predicted\_protein | BGC0001122 | Isoflavipucine\_biosynthetic\_gene\_cluster | 31 | 99.2766726944 | 228 | 1.8e-59 |
| ADY16699.1 | TqaJ | BGC0001142 | Tryptoquialanine\_biosynthetic\_gene\_cluster | 31 | 84.2676311031 | 224 | 2.6e-58 |
| AMR44276.1 | MFS\_multidrug\_transporter | BGC0001398\_c3 | Phomopsins\_biosynthetic\_gene\_cluster | 28 | 91.5009041591 | 222 | 1e-57 |
| BAE71313.1 | putative\_ABC\_transporter | BGC0000004 | Aflatoxin\_biosynthetic\_gene\_cluster | 29 | 80.650994575 | 208 | 1.5e-53 |
| BAD29973.1 | transporter\_protein | BGC0000676 | Aphidicolin\_biosynthetic\_gene\_cluster | 30 | 82.6401446655 | 204 | 2.8e-52 |
| AAS90069.1 | AflT | BGC0000010 | Aflatoxin\_biosynthetic\_gene\_cluster | 29 | 78.4810126582 | 204 | 2.8e-52 |
| ADU85991.1 | putative\_membrane\_transport\_protein | BGC0000165 | Tiacumicin\_B\_biosynthetic\_gene\_cluster | 30 | 76.3110307414 | 198 | 2.6e-50 |
| AAS90021.1 | AflT | BGC0000008 | Aflatoxin\_biosynthetic\_gene\_cluster | 29 | 80.650994575 | 196 | 5.9e-50 |
| AAS90092.1 | AflT | BGC0000006 | Aflatoxin\_biosynthetic\_gene\_cluster | 29 | 80.650994575 | 196 | 1e-49 |
| AHW57792.1 | PgaJ3 | BGC0000262 | Rabelomycin\_biosynthetic\_gene\_cluster | 27 | 93.8517179024 | 192 | 1.4e-48 |
| AFU65888.1 | DacR2 | BGC0000216 | Dactylocycline\_biosynthetic\_gene\_cluster | 29 | 77.034358047 | 190 | 7.2e-48 |
| XP\_002384861.1 | MFS\_multidrug\_transporter,\_putative | BGC0001234\_c2 | 4,4'-piperazine-2,5-diyldimethyl-bis-phenol\_biosynthetic\_gene | 27 | 78.4810126582 | 190 | 7.2e-48 |
| CAA09636.1 | putative\_transmembrane\_protein | BGC0000227 | Granaticin\_biosynthetic\_gene\_cluster | 34 | 76.6726943942 | 184 | 3e-46 |
| ACD39765.1 | major\_facilitator\_superfamily\_transporter | BGC0000077 | Hypothemycin\_biosynthetic\_gene\_cluster | 28 | 83.0018083183 | 183 | 6.7e-46 |
| ACD39756.1 | major\_facilitator\_superfamily\_transporter | BGC0000076 | Hypothemycin\_biosynthetic\_gene\_cluster | 28 | 83.0018083183 | 183 | 6.7e-46 |
| AAB36607.1 | toxin\_pump | BGC0000370\_c2 | HC-toxin\_biosynthetic\_gene\_cluster | 28 | 77.5768535262 | 176 | 1.1e-43 |
| AAQ08935.1 | putative\_membrane\_transporter | BGC0000224 | Fredericamycin\_biosynthetic\_gene\_cluster | 26 | 89.6925858951 | 175 | 1.4e-43 |
| CBF87265.1 | MFS\_toxin\_efflux\_pump\_(AflT),\_putative\_(AFU\_orthologue;\_AFUA\_1G12620) | BGC0000680\_c2 | Austinol/dehydroaustinol,\_locus\_1\_biosynthetic\_gene\_cluster | 28 | 79.0235081374 | 173 | 7e-43 |
| ADG86335.1 | transporter | BGC0000190 | A-74528\_biosynthetic\_gene\_cluster | 26 | 88.9692585895 | 172 | 1.2e-42 |
| ACP19369.1 | SaqJ1 | BGC0000267 | Saquayamycin\_Z\_/\_galtamycin\_B\_biosynthetic\_gene\_cluster | 27 | 88.2459312839 | 171 | 2e-42 |
| AEI98661.1 | CtcR | BGC0000209 | Chlortetracycline\_biosynthetic\_gene\_cluster | 28 | 74.321880651 | 161 | 3.6e-39 |
| CBF76046.1 | conserved\_hypothetical\_protein | BGC0001399 | Fellutamide\_B\_biosynthetic\_gene\_cluster | 26 | 69.801084991 | 159 | 1.4e-38 |
| EAQ86390.1 | hypothetical\_protein | BGC0001405 | Chaetoviridin\_/\_chaetomugilin\_biosynthetic\_gene\_cluster | 28 | 70.3435804702 | 153 | 5.7e-37 |
| AAS89998.1 | AflT | BGC0000007 | Aflatoxin\_biosynthetic\_gene\_cluster | 34 | 33.0922242315 | 121 | 2.4e-27 |
| AAS90046.1 | AflT | BGC0000009 | Aflatoxin\_biosynthetic\_gene\_cluster | 34 | 33.0922242315 | 121 | 3.1e-27 |
| AIL50170.1 | putative\_transport\_protein | BGC0000213 | Colabomycin\_biosynthetic\_gene\_cluster | 24 | 76.130198915 | 121 | 4.1e-27 |
| ADZ13560.1 | YtkR6 | BGC0000466 | Yatakemycin\_biosynthetic\_gene\_cluster | 24 | 77.2151898734 | 103 | 8.9e-22 |
| CAP12594.1 | elloramycin\_permease | BGC0000219 | Elloramycin\_biosynthetic\_gene\_cluster | 26 | 74.321880651 | 91 | 3.5e-18 |
| ADI24938.1 | VrtL | BGC0000168 | Viridicatumtoxin\_biosynthetic\_gene\_cluster | 26 | 29.1139240506 | 66 | 1.6e-10 |
